# Evaluating the Potential Bioactivity of a Novel Compound ER1626

**DOI:** 10.1371/journal.pone.0086509

**Published:** 2014-01-27

**Authors:** Lijun Wang, Yanyan Zeng, Tianling Wang, Hongyi Liu, Hong Xiao, Hua Xiang

**Affiliations:** 1 Nanjing Medical University, Affiliated Nanjing Brain Hospital, Nanjing, China; 2 Department of Pharmaceutical Chemistry, China Pharmaceutical University, Nanjing, China; Southern Illinois University School of Medicine, United States of America

## Abstract

**Background:**

ER1626, a novel compound, is a derivate of indeno-isoquinoline ketone. This study was designed to evaluate the biological activity and potential anti-tumor mechanism of ER1626.

**Method:**

MTT assay, scratch assay and flow cytometry were used to determine cell proliferation, cell migration and cell cycle distribution as well as cell apoptosis on human breast cancer MCF-7 cells and endometrial cancer Ishikawa cells. We also explored the antiangiogenic effect of ER1626 on HUVEC cells and chicken embryos. The expression of estrogen receptor protein was investigated with western-blot analysis.

**Results:**

ER1626 down-regulated the expression of estrogen receptor α protein and up-regulated β protein in MCF-7 and Ishikawa cells. The value of IC_50_ of ER1626 on MCF-7 and Ishikawa cells were respectively 8.52 and 3.08 µmol/L. Meanwhile, ER1626 decreased VEGF secretion of MCF-7 and Ishikawa cells, disturbed the formation of VEGF-stimulated tubular structure in HUVEC cells, and inhibited the angiogenesis on the chicken chorioallantoic membrane. Scratch assay revealed that ER1626 suppressed the migration of MCF-7, Ishikawa and HUVEC cells. In addition to induction tumor cell apoptosis, ER1626 arrested cell cycle in G1/G0 phase in MCF-7 cells and G2/M phase in Ishikawa cells.

**Conclusion:**

In conclusion, our results demonstrated that ER1626 has favorable bioactivities to be a potential candidate against breast cancer and angiogenesis.

## Introduction

Breast cancer affects one in eight women during their lives. Breast cancer kills more women in the United States than any cancer except lung cancer [1]. More than 40 000 women die from breast carcinoma every year and the incidence of this disease increases year by year. More and more confirmed cases bring the total to 130 000 annually on a global scale [2].

Estrogen hormone has been known to play an essential role in the carcinogenesis of breast tumor [3], [4]. It participates in the physiological and pathological regulation by binding to estrogen receptors (ERs). ERs exist in two stereotactic forms- ERα and ERβ. They are similar in their molecular architecture as other nuclear superfamily members, but they differ in tissue distribution and ligand-banding ability [5], [6], which underlies their diverse responses to estrogen. The balance between ERα and ERβ expression is important for normal estrogen function in the breast and other tissues. In ER-positive breast tissue, the ERα/ERβ ratio differs between benign and malignant disease with a predominance of ERα in malignant and ERβ in benign tissue [7]. ERα and ERβ also shows difference in tissue distribution and efficacy at various gene regulatory sites [5]. Lifetime exposure to estrogen is an important risk factor for patients with breast cancer which stimulates the proliferation of breast epithelial cells [4], [8]. The mechanism of estrogen action led to the design of antiestrogenic agents which have come to be known as selective estrogen receptor modulators (SERMs). They have been widely used in postmenopausal estrogen therapy in the past decades. SERMs manifest themselves as agonists in some estrogen target tissues while antagonists in others. Their selective traits are relative to several factors, including specific tissues, chemical structure, ER subtypes, co-regulators and intracellular signaling pathway, etc [9].

Two representative drugs, Tamoxifen (TAM) and Raloxifene, have proved to be effective in the treatment of breast cancer, especially for ER-dependent types. The observation from the Breast Cancer Prevention Trial of the National Surgical Adjuvant Breast and Bowel Project showed that TAM markedly reduced the risk of invasive breast cancer, particularly tumors with positive estrogen receptor status. In addition to that, TAM decreased lightly the incidence of fractures. However, TAM resulted in seriously high-risk endometrial cancer, stroke, pulmonary emboli, deep-vein thrombosis, and cataracts, primarily in women over 50 years old [10]–[13]. In the Study of Tamoxifen and Raloxifene (STAR) trial, findings revealed that Raloxifene was as effective as TAM in the prevention of invasive breast cancer without increasing the risk of endometrial carcinoma of uterus [14]. Raloxifene increased bone density in spine and femoral neck and lowered the rates of vertebral fractures [15]. Clinical benefits of Raloxifene always go with negative outcomes, which mainly involve seriously venous thromboembolism and fatal stroke. Other common adverse events cover hot flushes, leg cramps, peripheral edema, and gallbladder disease [16]–[19].

Vascular Endothelial Growth Factor (VEGF) is a prominent regulator of physiological angiogenesis and pathological angiogenesis involved in tumors [20]. It plays an important role in encouraging tumor growth mainly by stimulating the growth of tumor blood vessel and increasing vascular permeability [21]–[23]. VEGF is secreted by endothelial cells and various tumor cells, especially tumor cells in hypoxia area [20], [24], [25]. In this study, we tried to explore the antitumor effect of ER1626 by leaning VEGF secretion.

**Figure 1:**
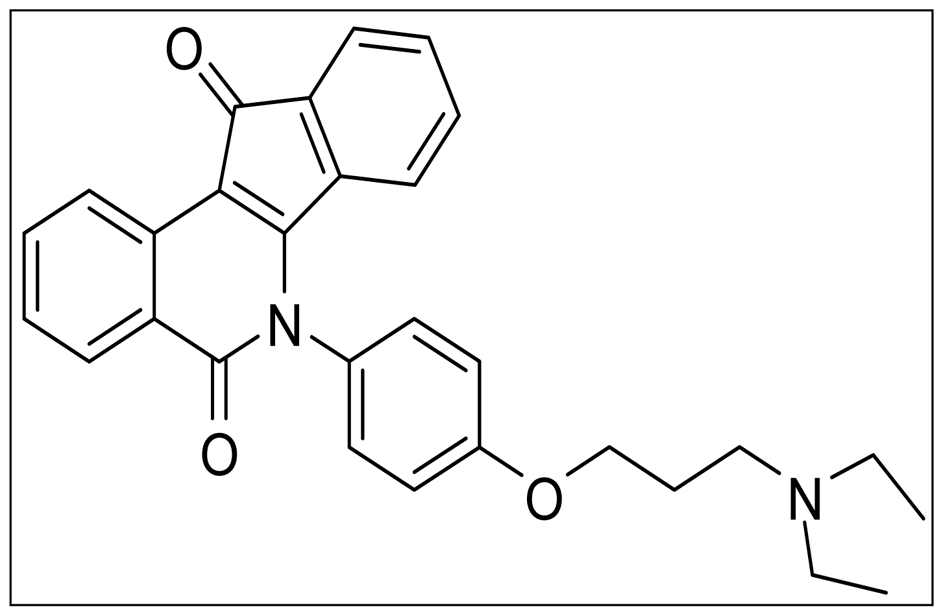
The chemical structure of ER1626.

The objective of our study was to investigate the *in vitro* and *in vivo* bioactivity and potential tumoricidal mechanism of a novel patent compound ER1626 [6-(4-(3-(diethyllamino) propoxy) phenyl)-5H-indeno [1, 2-c] isoquinoline-5, 11(6H)-dioneis] (Fig. 1) [26], [27].

## Materials and Methods

### Reagents

ER1626 was synthesized by China Pharmaceutical University. TAM, Raloxifene and [3-(4, 5-dimethyl thiazol-2-yl) -2, 5-diphenyltetrazolium bromide] (MTT) was purchased from Sigma-Aldrich (USA). Anannexin V-fluorescein Isothiocyanate (FITC) Apoptosis Kit and Cell Cycle Detection Kit were obtained from KeyGEN Biotechnology (Nanjing, China). Anti-ERα (ab16813) and Anti-ERβ (ab2746) antibody was purchased from Abcam Inc. Experiments were performed with 1×10^−2^ mol/L of DMSO-solute ER1626 stock solution, stored in −20°C, diluting to target concentration in the conditioned medium.

### Ethics Statement

This study was approved by the Committee on the Medical Ethics of Nanjing Brain Hospital Affiliated to Nanjing Medical University (Permit Number: 2012KY024).

### Cell Lines and Cell Culture

Ishikawa, HUVEC and estrogen receptor-positive MCF-7 cell lines were obtained from KeyGEN Biotechnology. Cells were maintained in RPMI-1640(Gibco, Carlsbad, CA) medium containing 10% fetal bovine serum (Gibco, Carlsbad, CA) and 1% penicillin-streptomycin at 37°C in 5% CO_2_, unless otherwise mentioned.

### Western Blot Analysis

MCF-7 and Ishikawa cells were treated with ER1626 (10^−7^, 10^−6^ and 10^−5^ mol/L) for 24 h in 6-well culture dishes and then lysed in ice-clod cell lysis buffer supplemented with protease inhibitor cocktail (Promega, Madison, WI, USA). Total protein extracts were kept in boiling water for 3 min and separated by gel electrophoresis and then transferred to Immuno-Blot™ PVDF membrane (Millipore). Non-specific binding was blocked in 5% skimmed milk for 2 h. PVDF membrane was incubated with primary murine antibody, anti-ERα (1∶1 000), anti-ERβ (1∶1 000) or antibody against β-actin (1∶400) overnight at 4°C. The membrane was then washed and incubated with a HRP-conjugated goat anti-mouse secondary antibody (1∶10 000) for 1 h at room temperature. Immunoreactive bands were detected using ECL chemiluminescence reagents (Pierce, USA) and visualized by the Gel Image Analysis software. Relative expression of ERα and ERβ was determined by densitometry.

### Cell Proliferation Assay

The growth inhibitory effect was determined by MTT assay [28]–[31]. Briefly, MCF-7 and Ishikawa cells were seeded into 96-well plates until approximately 80% confluency and incubated with various concentrations of ER1626 (10^−7^, 10^−6^, 10^−5^ or 10^−4^ mol/L) or 0.1% DMSO -vehicle for 48 h, using TAM and Raloxifene as reference substance. A 20 µl aliquot MTT solution (5 mg/mL) was added in each well and incubated for another 4 h. Then, supernatants were removed, followed by the addition of 150 µl DMSO in each well. The optical density (O.D) was read with Microquant (Biotech, USA) at 570 nm. Identical concentrations were tested in triplicate and the assay performed three times. The inhibition ratio of cell proliferation was calculated following the Formula 1.
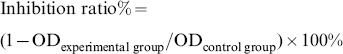



### VEGF Production Detected by ELISA Kit

MCF-7 and Ishikawa cells were cultured in 6-well dishes overnight. The culture medium was replaced with 5% FBS medium containing various concentrations of ER1626 (10^−9^, 10^−8^, 10^−7^, 10^−6^ and 10^−5^ mol/L). After 24 h, the density of VEGF in the conditioned medium was measured using a commercial ELISA kit (Boster, Wuhan, China) following manufacture’s instruction.

### Tube Formation

A 96-well plate was coated with 80 µl of Matrigel (Becton-Dickinson, Bedford, MA) per well to form a reconstituted basement membrane matrix at 37°C for 30 min. HUVEC cells were harvested, diluted in a density of 150 000 cells/mL in ECM medium containing 5% FBS and 10 ng/mL of VEGF(Boster, Wuhan, China). A volume of 100 µl re-suspended cells were added onto the gelled matrix and treated with ER1626 (10^−7^, 10^−6^ and 10^−5^ mol/L).

### Chicken Embryo Chorioallantoic Membrane (CAM) Assay

Antiangiogenic activity of ER1626 was assayed using a modified chicken chorioallantoic membrane assay [32]. Fertilized chicken eggs were incubated at 37°C for 5 d. A window was carefully created through the pore of egg and then the shell membrane was detached gently. After that, the window was covered with plastic wrap to form a fake-pore and the egg was adapted for one day more in the incubater. Filter paper disks (5×5 mm) saturated with 10 µl of ER1626 (5×10^−4^ or 5×10^−3^ mol/L) or 2-Methoxyestradiol (5×10^−3^ mol/L) or 0.1% DMSO were placed on CAMs. Eggs were then incubated for another 2 d and CAMs under paper disks were harvested and photographed.

### Scratch Assay

MCF-7, Ishikawa and HUVEC cells were respectively seeded into 6-well culture plates overnight. Cells were treated with different does of ER1626 (10^−7^, 10^−6^ and 10^−5^ mol/L) before scraping the confluent cell monolayer in a straight line to create ‘scratches’. Images of five randomized visions of each scratch were captured with a phase-contrast microscope at 0, 24 and 48 h (extending to 72 h for HUVEC cells). The cell migration rate was calculated with mean width following the Formula 2.

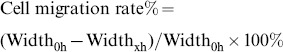



### Cell Cycle Assay

MCF-7 and Ishikawa cells were respectively planted into 6-well dishes, cultivated in serum-free culture medium for 24 h, and then treated with ER1626 solution (10^−6^ mol/L). Following the manufacturer’s protocol, cells were collected and processed with Cell Cycle Detection Kit at 6, 12, 24 and 48 h. Results were determined using BD FACSCalibur flow cytometer (FCM) (488 nm excitation, 610 nm emission). The result was analyzed by CellQuest software (Becton Dickinson, CA).

### Apoptosis Assay

Tumor cells were grown to 80% confluency in 6-well plates, treated with ER1626 (10^−7^, 10^−6^ and 10^−5^ mol/L), and incubated for 24 h. Treated cells were processed with annexin V-FITC Apoptosis kit (KeyGEN, Nanjing, China) and were detected the distribution of cell state with FCM according to operation instruction.

### Statistical Analysis

Data were analyzed by Student’s *t* test and one-way ANOVA using Statistical Package for Social Science (SPSS12.0, Chicago, IL). Data were expressed as the mean ± standard deviation (SD). Differences are considered to be significant at *p<0.05*.

## Results

### Effect of ER1626 on ERα and ERβ Protein Levels

**Figure 2:**
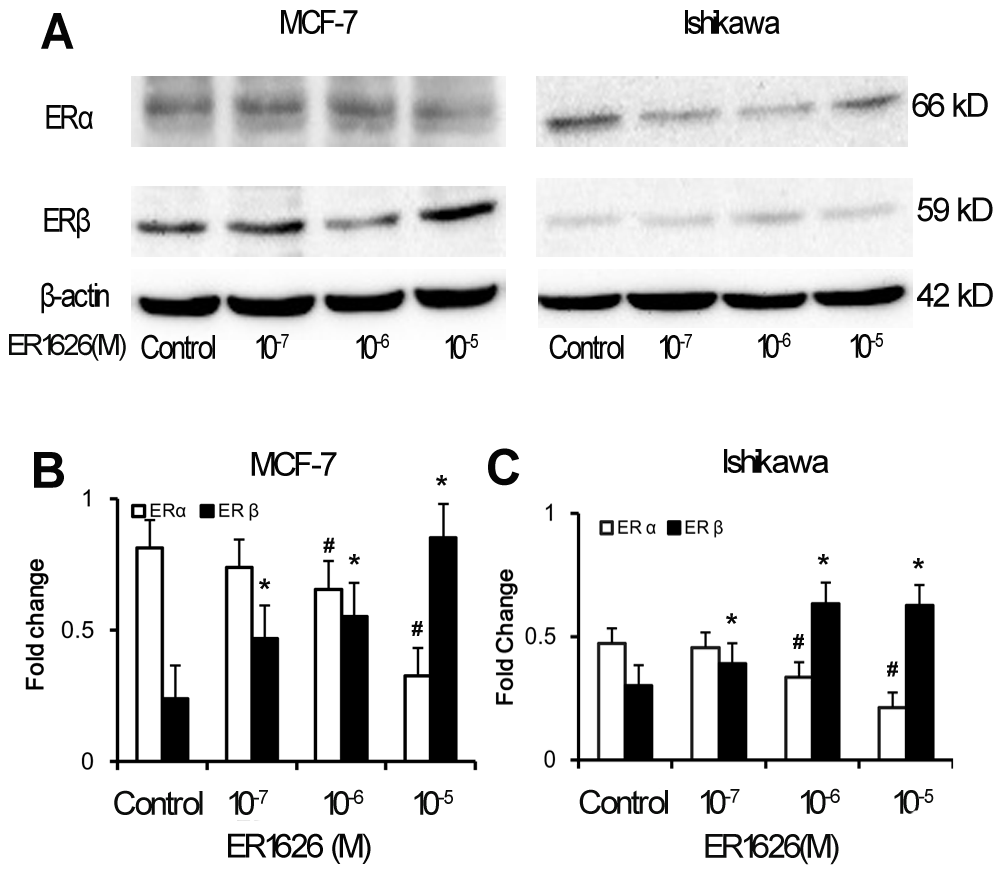
The expression of ERα and ERβ protein in ER1626-treated MCF-7 and Ishikawa cells. **A**. Cells were respectively in 6-well plates and maintained in specific medium supplemented with 5% FBS for 24 h prior to incubation with ER1626 (10^−7^, 10^−6^ and 10^−5^M) or vehicle for another 24 h. Treated cells were lysed in RIPA buffer and cell lysate was electrophoresed. Immunoblotting was performed for ERα, ERβ and the loading control β-actin. **B** The intensity of the band of ERs protein was normalized and expressed as relative fold change in MCF-7 cells. **C** The intensity of ERs protein was expressed in Ishikawa cells.*p<0.05,^ #^ *p*<0.05 compared with their corresponding control.

After being treated with ER1626 for 24 h, total protein of MCF-7 and Ishikawa cells were respectively extracted with protein lysis buffer and examined by western blot. Immunoblotting revealed that at the concentration of 10^−6^ and 10^−5^ mol/L of ER1626 reduced ERα protein level but increased ERβ protein level when compared to the controls (*p<0.05*) on both MCF-7 and Ishikawa cells (Fig. 2).

### Effect of ER1626 on Proliferation of MCF-7 and Ishikawa Cells

**Figure 3:**
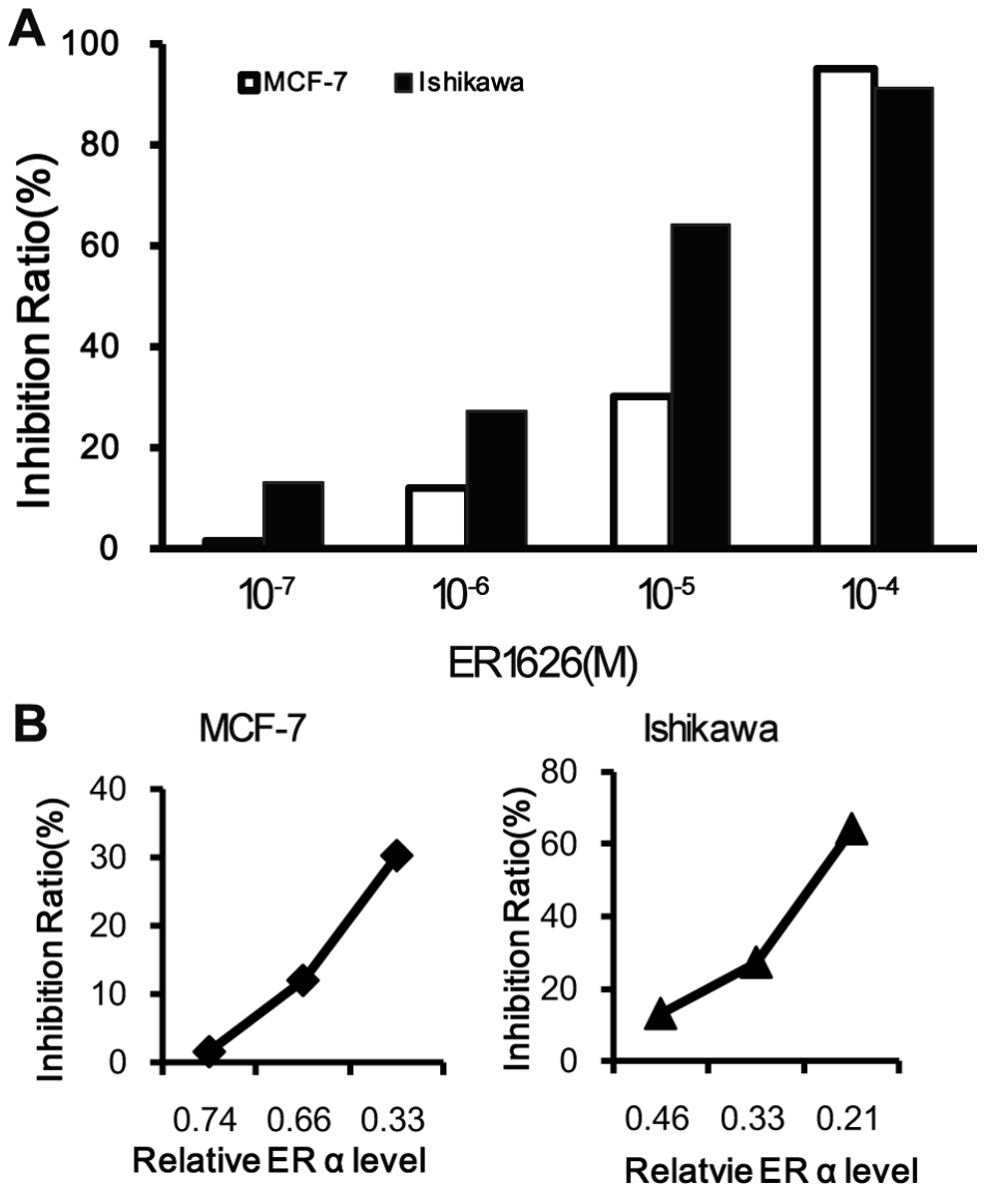
Inhibitory effect of ER1626 on the proliferation of MCF-7 and Ishikawa cells. **A** Cells were seeded into 96-well plates and incubated overnight, then added MTT solution per well after incubation with 100 µl of ER1626 solution (10^−7^, 10^−6^, 10^−5^ or 10^−4^M) for 48 h. Medium was replaced with 150 µL DMSO. The optical density was measured at 570 nm. The growth inhibition ratios in the histogram were calculated following the formula: **B** the proliferation inhibition ratio versus the relative ERα level in ER1626-treated MCF-7 and Ishikawa cells.

ER1626 suppressed the cell proliferation of the tested tumor cells in a dose-dependent manner. At the concentration of 1×10^−4^ mol/L, the growth inhibition ratios of ER1626 exceeded 90% on the two assayed cell lines. The IC_50_ of ER1626 on MCF-7 and Ishikawa cells was 8.52×10^−6^ mol/L and 3.08×10^−6^ mol/L respectively. In contrast, the IC_50_ of TAM on MCF-7 and Ishikawa cells was 3.14×10^−6^ mol/L and 4.59×10^−6^ mol/L, and Raloxifene was 6.56×10^−6^ mol/L and 7.50×10^−6^ mol/L, respectively. The height of each bar graph presents the inhibition ratio of the corresponding concentration (Fig. 3A). Comparing and analyzing ‘the inhibition ratio’ with ‘the relative ERα protein level’, the inhibition ratio rised with the decreased ERα level in both MCF-7 and Ishikawa cells (Fig. 3B).

### Effect of ER1626 on VEGF Production

**Figure 4:**
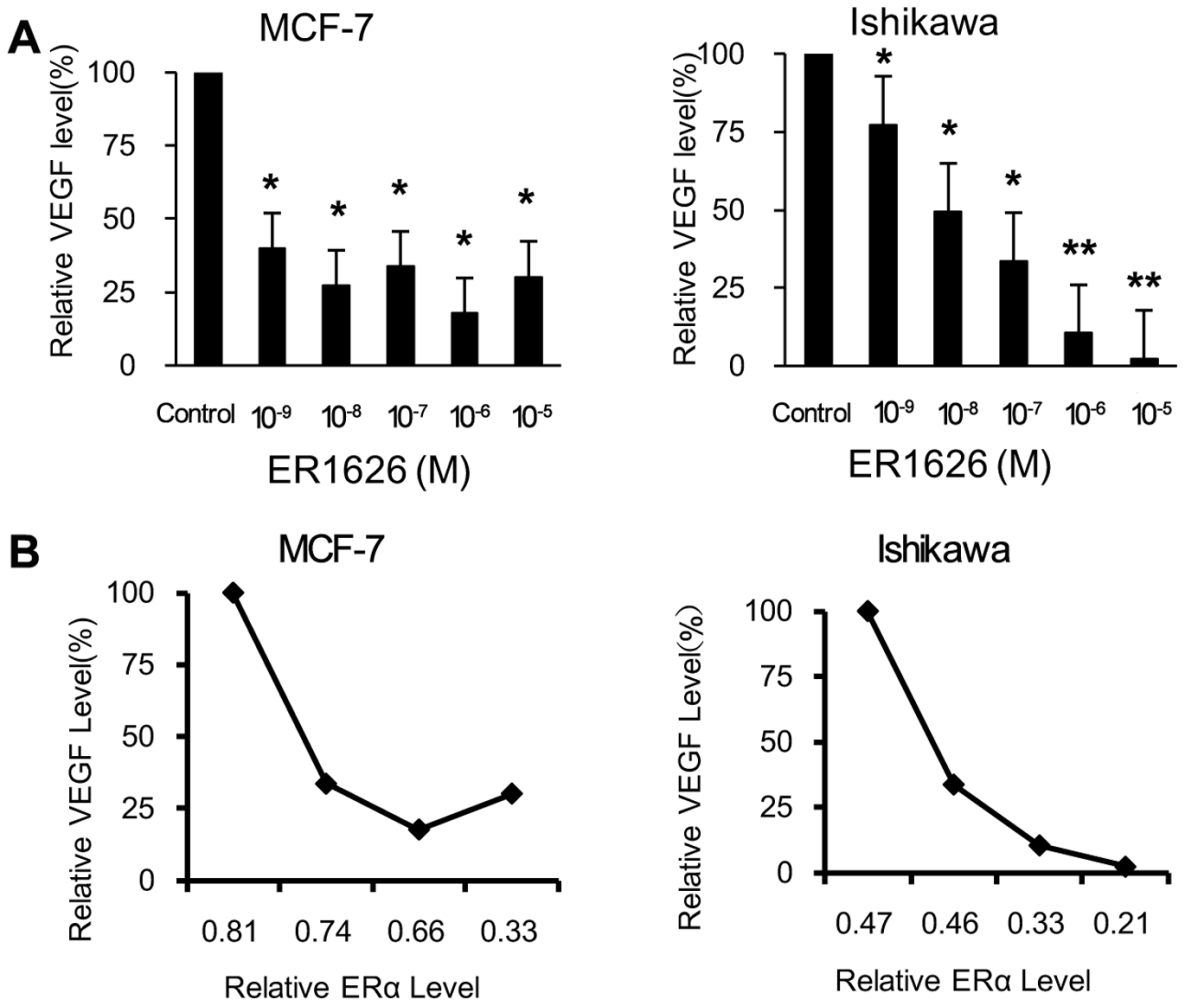
Reduction of VEGF secreted by MCF-7 and Ishikawa cells. **A** MCF-7 and Ishikawa cells were planted respectively in 6-well dishes overnight and incubated with ER1626 (10^−9^, 10^−8^, 10^−7^, 10^−6^ or 10^−5^M) or control for 24 h. Elisa kit was employed to detect the VEGF production in the cultured medium. Inhibition ratio was expressed as the percentage of VEGF product in treated cells to those of the control cells (100%). **B** The relative VEGF level in ER1626-treated cultured system versus the relative ERα level in ER1626-treated MCF-7 and Ishikawa cells. *p<0.05, **p<0.01 compared with the control.

We examined the VEGF production in the supernatant of MCF-7 and Ishikawa cell medium by ELISA kit and assessed the effect of ER1626 treatment on VEGF secretion. In both MCF-7 and Ishikawa cultured system, dramatic shrink of VEGF secretion was observed in ER1626-treated groups compared with their control (untreated) groups. The results were expressed as the percentage of VEGF product in treated cells to those of the control cells (100%) (Fig. 4A). Differences between the control and tested groups were statistically significant (*p<0.05*). In general, VEGF secretion descended with the decreasing of the relative ERα protein level in MCF-7 and Ishikawa cells (Fig. 4B).

### Effect of ER1626 on HUVEC Cell Migration and Tube Formation

**Figure 5:**
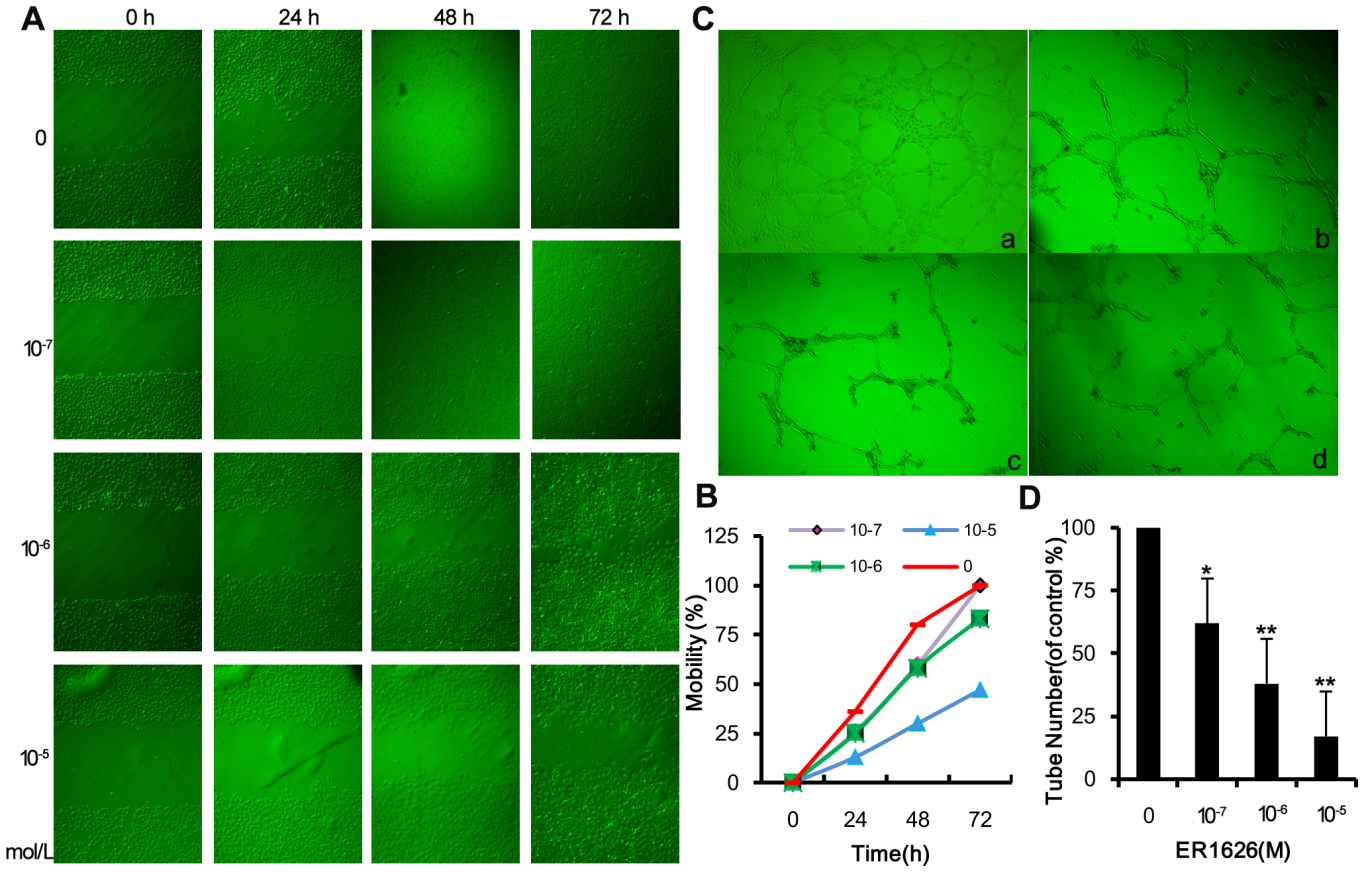
Effect of ER1626 on HUVEC cell migration and tube formation. HUVEC cells were cultured in 6-well plates overnight, creating a straight scratch on each confluent monolayer and incubating with ER1626 (10^−7^, 10^−6^ and10^−5^M) or vehicle. After 0, 24, 48 and 72 h, images of five randomized vision of scratches were captured and the width of scratches was measured. Cell migration ratio was calculated following the formula: Cell migration ratio was presented in the curve diagram. **A** Images of the scratch in HUVEC cells; **B** The curve diagram of HUVEC cell migration. HUVEC cells were re-suspend in 96-well plate previously coated with 80 µl of commercial matrigel per well and incubated with ER1626 (10^−7^, 10^−6^ and 10^−5^M) for 8 h. Five randomly pictures of the enclosed networks of complete tubes were photographed under the microscope and the tube number was reckoned. The inhibition ratio of ER1626 was counted as the percentage of intact networks number in treated cells to those of the control cells (100%) and shown in the bar graph. **C** Images of VEGF-stimulated tube formation in HUVEC cells. a Control; b ER1626(10^−^ M); c ER1626(10^−6^M); d ER1626(10^−5^M); **D** Inhibition ratio of ER1626 on tube formation in HUVEC cells. *p<0.05, **p<0.01 compared with the control.

In vitro scratch assay was conducted to evaluate the effect of ER1626 on HUVEC migration. In the concentration of 10^−7^, 10^−6^ and 10^−5^ mol/L, ER1626 showed markedly inhibitory effect on cell migration in a dose-dependent way within 48 h (Fig. 5A). At the density of 10^−6^ and 10^−5^ mol/L, ER1626 still played a role in cell migration after 72 h treatment. Cell migration rates were calculated following the formula 2(Fig. 5B).

The ability of ER1626 to prevent vessel assembly was analyzed by seeding HUVEC with ER1626 or vehicle onto basement membrane matrix layers. Upon being planted, HUVEC cells rapidly attached, aligned, and formed capillary-like tubules on top of the basement membrane matrix. After incubation for 8 h, the enclosed networks of complete tubes from five randomly chosen fields were counted and photographed under the microscope (Fig. 5C). The percentage of intact networks amount in treated cells to those of the control cells (100%) was shown in Fig. 5D. The results indicated that ER1626 prevented VEGF-stimulated tube formation on HUVEC cells in a time and dose-dependent manner. Significant differences between the control and treatment groups were observed (*p<0.05*).

### ER1626 Inhibits the Angiogenesis of Chicken Embryos

**Figure 6:**
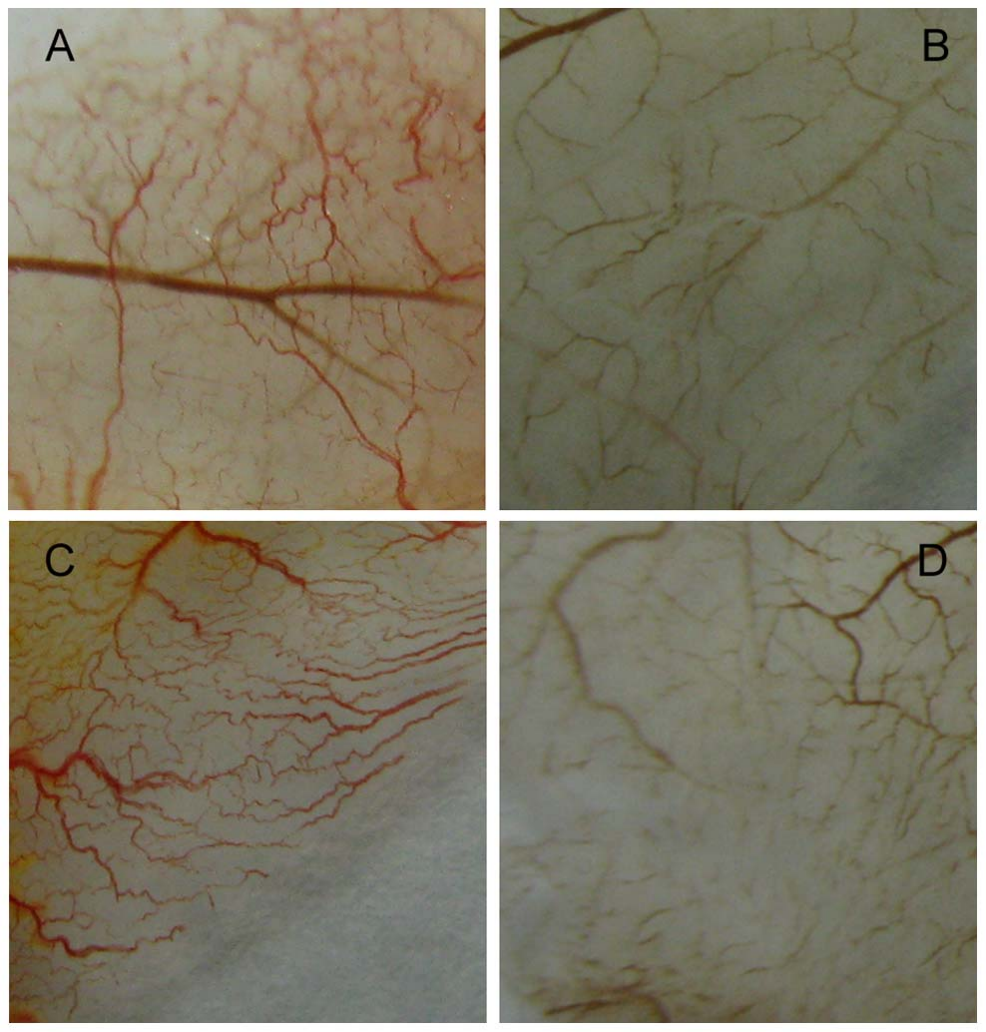
ER1626 inhibits the angiogenesis of chick embryos. A window was carefully created through the pore of 5-day chicken embryo. The window was covered with plastic wrap to form a fake-pore after the shell membrane on CAM was detached gently. Chicken embryos were adapted for one day more in incubater. Filter paper disks (5×5 mm) saturated with 10 µl of ER1626 (5×10^−4^ or 5×10^−3^M) or 2-Methoxyestradiol (5×10^−3^M) or 0.1% DMSO were lightly placed on CAMs. They were then incubated for 48 h and CAMs under paper disks were harvested and photographed. Compare the blood vessel on CAMs. **A** Control (0.1% DMSO); **B** 2-ME2 (16 µg); **C** ER1626 (2.3 µg); **D** ER1626 (23 µg).

We explored the potential antiangiogenic activity of ER1626 in the chick embryos. ER1626 showed its antiangiogenic effect to vessels on chicken embryo chorioallantoic membrane. Neovascular was normal on CAMs in the control group (Fig. 6A). ER1626 at 2.3 µg (Fig. 6C) per egg for 48 h showed a notable restraint, whereas 23 µg (Fig. 6D) per egg ER1626 drastically inhibited neovascularization of the CAM, accompanied by a lack of prominent vessel networks. The inhibition of 2ME2 was also shown (Fig. 6B). These results demonstrate that ER1626 is able to suppress angiogenesis in embryos.

### Effect of ER1626 on Migration of MCF-7 and Ishikawa Cells

**Figure 7:**
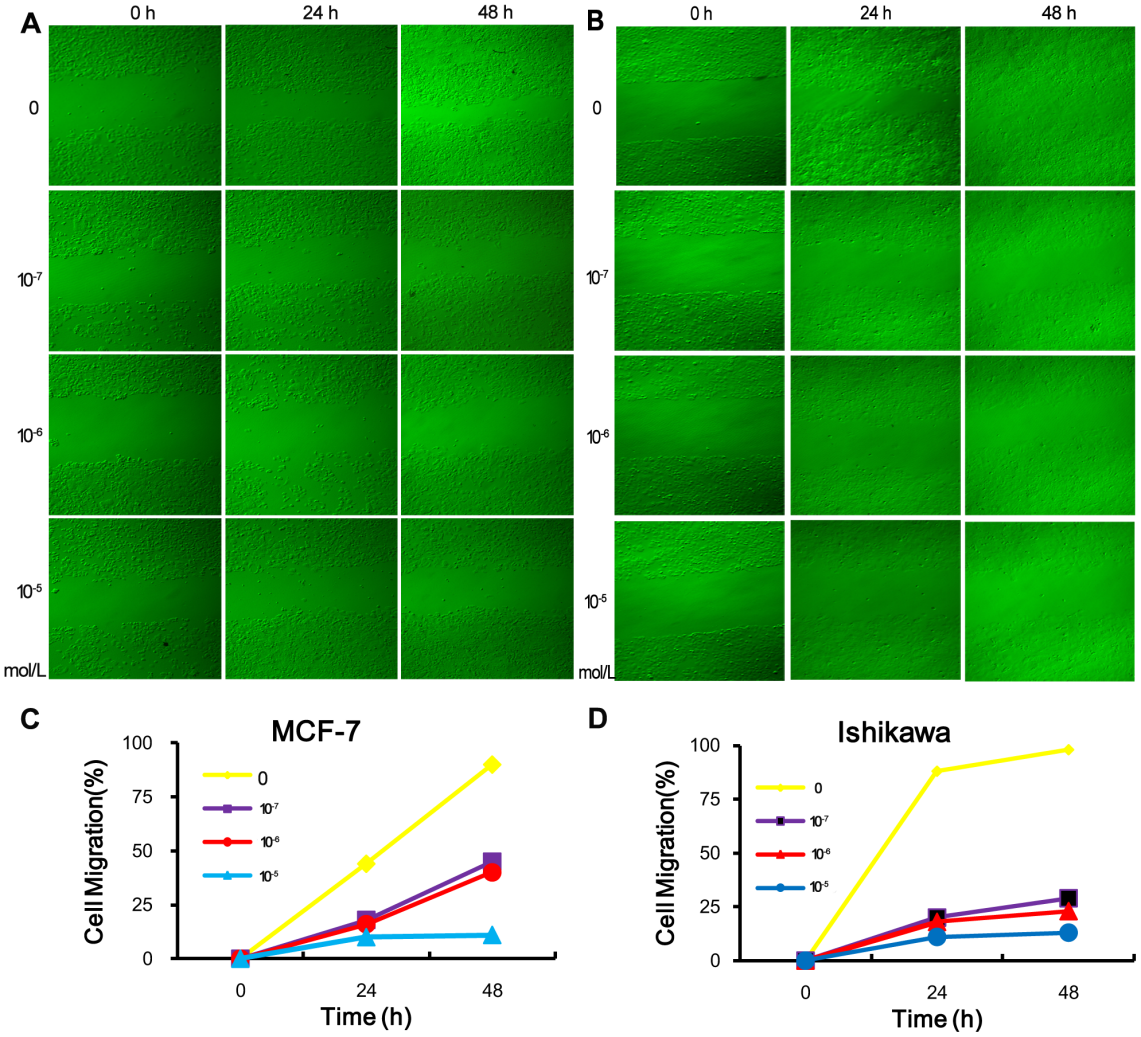
In vitro scratch assay of MCF-7 and Ishikawa cells treated with ER1626. Cells were planted into 6-well plates and scratches were created on the confluent monolayer before incubation with different concentrations of ER1626 (10^−7^, 10^−6^ and10^−5^M) or vehicle. After 0, 24 and 48 h, images of scratches were captured and the width of scratches was measured. Cell migration ratio was calculated following the formula: **A** Images of scratches of MCF-7 cells; **B** Images of scratches of Ishikawa cells; **C** The curve diagram of MCF-7 cells**; D** The curve diagram of Ishikawa cells.

The results of the scratch assay conveyed that ER1626 had prominently inhibitory effects on MCF-7 and Ishikawa cells concentration-dependently, ranging in concentration from 10^−7^ to 10^−5^ mol/L (Fig. 7A and 7B). In all experimental groups, ER1626 could inhibit the cell migration within 48 h. After exposure to 10^−5^ mol/L of ER1626 for 48 h, the mobility of MCF-7 and Ishikawa cells were less than 10%. Cell migration rates were calculated following formula 2(Fig. 7C and 7D).

### Effect of ER1626 on the Cell Cycle

**Figure 8:**
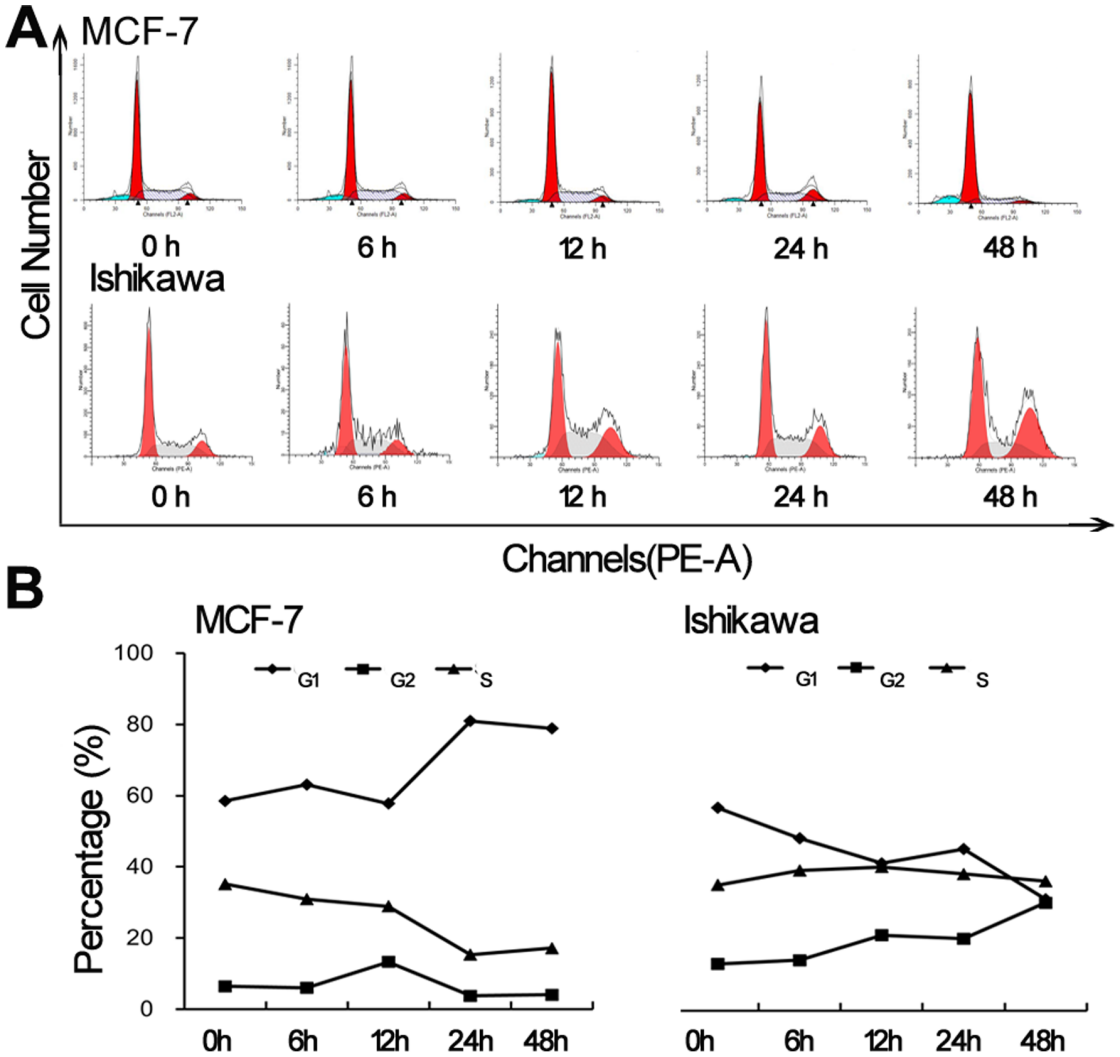
Flow cytometric analysis of cell cycle distribution in ER1626-treated MCF-7 and Ishikawa cells. Cells were incubated with ER1626 (10^−6^M) within various time intervals (6, 12, 24 and 48 h) and processed with Cell Cycle Detection Kit before being detected in flow cytometry (488 nm excitation, 610 nm emission). Data were analyzed by CellQuest software. The result was expressed as the varied proportion of the cell population in the G1/G0, S and G2/M phase in the line graph. **A** Cell distribution of ER-1626-treated MCF-7 and Ishikawa cells; **B** Variation of the percentage of cell population in whole cell cycle.

Representative cell-cycle changes were detected in two tumor cells after exposure to ER1626 within different durations (Fig. 8A). Being treated with ER1626 for 48 h, there was a comparable increase in the population in G1/G0 phase accompanied by a decreased cell number in S phase for MCF-7 cells, and a significant increase in G2/M phase with the S phase remained constant for Ishikawa cells (Fig. 8B). ER1626 may arrest the division cycle of MCF-7 and Ishikawa cell in different way.

### ER1626 Inductive Effect on Cell Apoptosis in MCF-7 and Ishikawa

**Figure 9:**
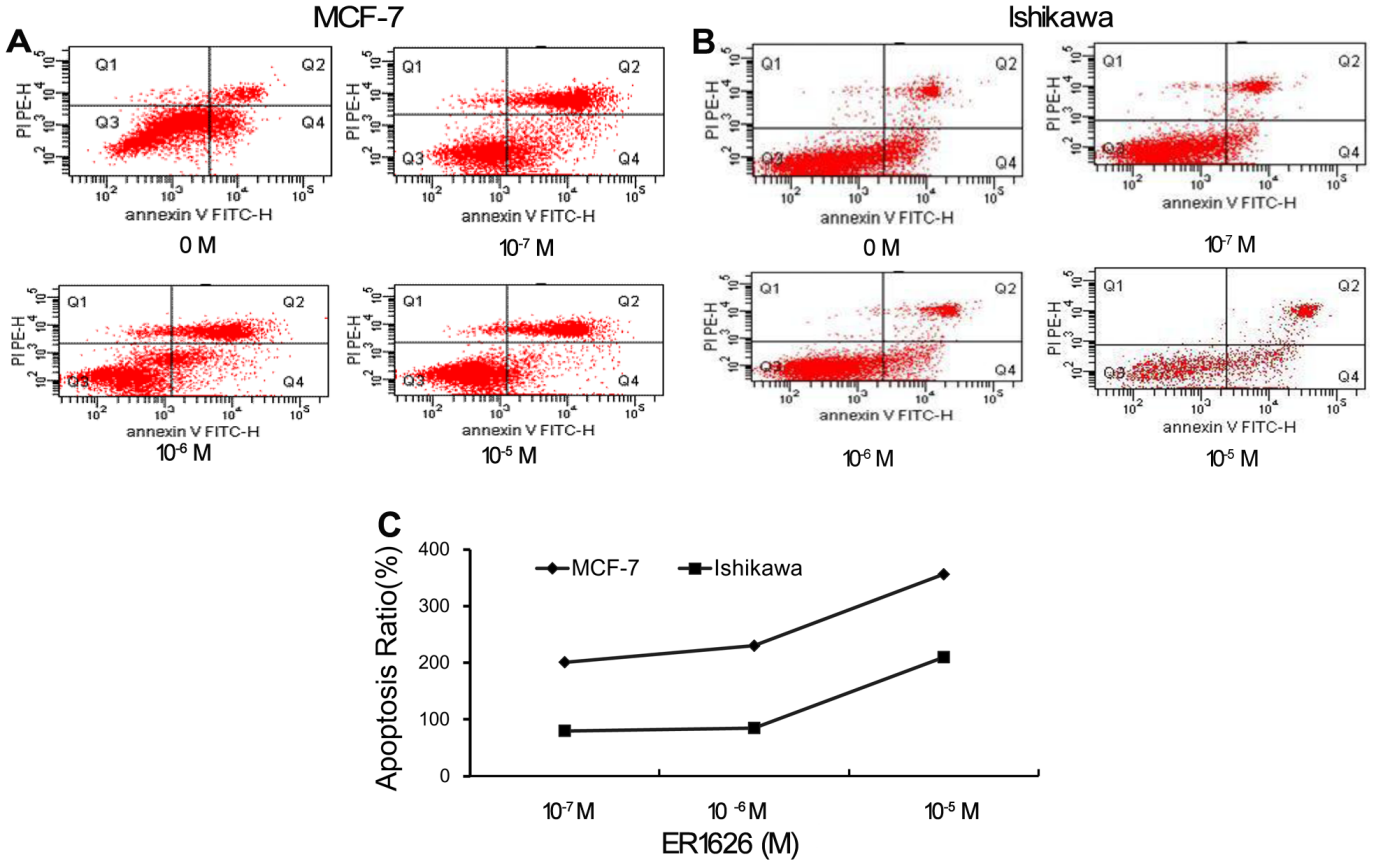
ER1626 inductive effect on cell apoptosis of MCF-7 and Ishikawa. Cells were grown in 6-well plates and apoptosis assay were performed following incubation with ER1626 (10^−7^, 10^−6^ and10^−5^M) for 24 h. Treated cells were processed with annexin V-FITC Apoptosis kit and analyzed in flow cytometery. **A** Typical pictures of the apoptotic cells in MCF-7; **B** Typical apoptosis pictures of Ishikawa cells; **C** Apoptosis ratios (the number of apoptotic cells in treatment groups to that of control group).

The Annexin V-FITC/PI double-staining analysis demonstrated that the population of apoptotic cells aggravated with the increment of ER1626 in MCF-7 and Ishikawa cells (Fig. 9A and 9B). There was a positive correlation between apoptosis ratio and ER1626 dose between 10^−7^ and 10^−5^ mol/L. Response to the increment of ER1626, the apoptosis ratio increased notably in MCF-7 and Ishikawa cells (Fig. 9C). ER1626 induced apoptosis of MCF-7 and Ishikawa cells in a dose-dependently manner.

## Discussion

ER1626 is a derivate of indeno-isoquinoline ketone. When MCF-7 and Ishikawa cells were exposed to designated dose ER1626 for 24 h, ERα protein level was down-regulated and ERβ protein was up-regulated moderately in MCF-7 and Ishikawa cells. The change between the ERα and ERβ supply an exciting possibility to improve the benefit-risk profile in MCF-7 and Ishikawa cells [7], [33], [34]. From previous studies, the ratio of the two ER subtypes may be the key factor as to how the ERs regulate the different cellular functions [35], and ERβ increases the efficacy by suppression on cell proliferation, cell apoptosis and cell cycling in breast cancer cells [36]–[38]. In our study, treatment with ER1626, ERβ protein was raised in MCF-7 and Ishikawa cells, which suggested the mechanism of anti breast cancer and endometrial cancer of ER1626 was associated with the increment of ERβ protein.

Previous study demonstrates that VEGF is one of target genes for ERs and contributes to breast cancer progression [39]. In our experiment, ER1626 reduced the production of VEGF and cut off partly the source of VEGF in MCF-7 and Ishikawa cells. The VEGF secretion declined when ERα level decreased in ER1626-treated MCF-7 and Ishikawa cells, this phenomenon may be explained due to the reduction of ERα level.

It is well-known that blood supply is an essential part of tumor survival and the integrated blood vasculature transports ample nutriment and metabolite for tumors. Angiogenesis is necessary for invasive tumor growth and needless metastasis as well as an important point for controlling tumor progression. Antiangiogenic therapy and neovascularization inhibition is a promising approach to the treatment of cancer. In our study, we found that ER1626 could inhibit markedly the formation of the VEGF-stimulated tubular structure of HUVEC cells. ER1626 also prevented the migration of HUVEC cells and blocked the tubule-like structure on HUVEC cells, which strongly indicated that ER1626 had the potential to block the fundamental requirements of tumor and attack tumor blood supply system. Actually, ER1626 even suppressed the new vessels of chick embryos. It is reported that the secreted VEGF from hypoxic tumor cells potently encourage tumor angiogenesis [40]. Reduction of VEGF secretion and inhibition of endotheliocyte migration could be combined with crippling tumor angiogenesis to destroy tumors *in vivo*. Our findings suggested that ER1626 probably by inhibition of ERα, thereby decreasing VEGF secretion, ultimately produced resistance to angiogenesis.

Tumor growth is a result of counterbalance between cell loss and proliferation. Compared with TAM, ER1626 shown comparative inhibitory effect on MCF-7 and Ishikawa cells. Our experiment validated that ER1626 not only blockaded the cell proliferation but also induced their apoptosis noticeably in MCF-7 and Ishikawa cells. Furthermore, ER1626 arrested cell cycle of MCF-7 cells by elevating numbers of cells in G0/G1 phase and decreasing number in S phase, which is to similar to TAM [41]. In a different way, the cycle of Ishikawa cells were arrested in G1/M phase. Cell proliferation suppression, cell cycle arrest and induction of cell apoptosis, these signatures may be explained by the up-regulated ERβ protein level in assayed cell lines [36], [38]. In other hand, there was a good effect relationship between ERα protein level and the ratios of cell proliferation in MCF-7 and Ishikawa cells, the rate of cell proliferation dose-dependently decreased accompanied by the lower of ERα protein level. We also conducted the scratch assay on MCF-7 and Ishikawa cells. The experimental results demonstrated that ER1626 inhibited the assayed cell migration.

In this paper we have described and analyzed the bioactivity and potential antitumor mechanism of ER1626, which indicates that ER1626 has characteristics of potential anti-breast tumor as well as anti-angiogenic activity. ER1626 not only down-regulated ERα protein level in breast cancer MCF-7 cells but also reduced ERα expression in human endometrial cancer Ishikawa cells. In addition, treatment with ER1626, ERβ protein was all up-regulated in MCF-7 and Ishikawa cells. What’s more, ER1626 exerted negative role on proliferation of tumor cells, arrest cell cycle and induced cancer cell apoptosis. ER1626 illustrates its strong point as a potential candidate of selective estrogen receptor modulator.
